# Therapeutic Algorithm for Use of Melatonin in Patients With COVID-19

**DOI:** 10.3389/fmed.2020.00226

**Published:** 2020-05-15

**Authors:** Russel J. Reiter, Pedro Abreu-Gonzalez, Paul E. Marik, Alberto Dominguez-Rodriguez

**Affiliations:** ^1^Department of Cell Systems and Anatomy, UT Health San Antonio, San Antonio, TX, United States; ^2^Department of Physiology, Faculty of Medicine, University of La Laguna, San Cristóbal de La Laguna, Spain; ^3^Division of Pulmonary and Critical Care Medicine, Eastern Virginia Medical School, Norfolk, VA, United States; ^4^Department of Cardiology, Hospital Universitario de Canarias, Santa Cruz de Tenerife, Spain; ^5^Facultad de Ciencias de la Salud, Universidad Europea de Canarias, Santa Cruz de Tenerife, Spain; ^6^CIBER de enfermedades CardioVasculares (CIBERCV), Madrid, Spain

**Keywords:** melatonin, COVID-19, SARS-CoV-2, treatment-drug, prevention & control

## Abstract

The coronavirus, COVID-19, has infected hundreds of thousands and killed tens of thousands of individuals worldwide. This highly infectious condition continues to ravage the world population and has yet to reach it peak infective rate in some countries. Many conventional drugs including hydroxychloroquine/chloroquine, lopinavir, remdesivir, etc., have been repurposed as treatments for this often deadly disease, but there is no specifically-designed effective drug available; also, the drugs mentioned have significant side effects and their efficacy is unknown. New drugs and vaccines are being designed as COVID-19 treatment, but their development and testing will require months to years. Time is not a luxury that this crisis has. Thus, there is a serious unmet need for the identification of currently-available and safe molecules which can be used to slow or treat COVID-19 disease. Here, we suggest melatonin be given consideration for prophylactic use or treatment alone or in combination with other drugs. Melatonin's multiple actions as an anti-inflammatory, anti-oxidant, and anti-viral (against other viruses) make it a reasonable choice for use. Melatonin is readily available, can be easily synthesized in large quantities, is inexpensive, has a very high safety profile and can be easily self-administered. Melatonin is endogenously-produced molecule in small amounts with its production diminishing with increased age. Under the current critical conditions, large doses of melatonin alone or in combination with currently-recommended drugs, e.g., hydroxychloroquine/chloroquine, to resist COVID-19 infection would seem judicious.

## Introduction

In the past 20 years, two coronavirus epidemics that originated in China caused large-scale pandemics that involved over 20 countries leading to ~8,000 cases and 800 deaths. In 2002 the Middle East respiratory syndrome coronavirus produced 2,500 cases with infection and caused 800 deaths. The coronavirus disease 2019 (COVID-19) is highly contagious and has quickly spread globally (1). Using mathematical models, the attack rate of COVID-19 suggests an estimate of reproduction (R0) to be 2–3 indicating that 60% of the population will likely become infected (2). As of March 31, 2020 there have been 777,798 cases of COVID-19 reported worldwide, with 37,272 fatalities (3).

The cardinal symptoms of COVID-19 are cough, fever, and shortness of breath. These symptoms appear 2–14 days after infection (4, 5). The clinical picture varies from pausymptomatic to more serious clinical situations such as severe respiratory failure, sepsis, shock, and multiple organ dysfunction syndrome (6). Currently, there is no specific treatment for COVID-19, so drugs need to be developed or reused to end the pandemic. The World Health Organization has launched a clinical trial called SOLIDARITY to investigate 4 potential treatments: lopinavir and ritonavir plus interferon-beta, lopinavir and ritonavir, chloroquine/hydroxychloroquine, and remdesivir (7). The medical profession has quickly realized that there is no cure for this disease and vaccines will not be available for several months. This leaves a large unmet need for safe and effective treatments for COVID-19-infected patients. Obviously, there is a very urgent need for a cheap, viable, and readily available treatment such as melatonin (8).

Melatonin is synthesized from tryptophan in the pineal gland and by almost all the organs of the body, since its production is associated with mitochondria. It is noteworthy that high levels of melatonin play positive roles in health and aging. Melatonin, a well-known chronobiotic, is also a promising adjunctive drug for viral infections due to its anti-inflammatory, antiapoptotic, immunomodulatory, and powerful antioxidant properties (8). Herein, we review the current evidence for a role of melatonin as a COVID-19 treatment. Since the clinical data is very limited, we propose the use of melatonin in patients with COVID-19 to reduce morbidity and mortality.

## Rationale for Melatonin Use in Patients With COVID-19

Little is known about the crucial factors of disease severity and immune alteration produced by COVID-19 infection in humans (9). Cytokines and chemokines play important roles in immunity, demonstrating that an exaggerated immune response causes lung damage and a greater probability of death. In individuals infected with COVID-19, interleukin (IL)−10, 6 and tumour necrosis factor (TNF) -α are increased during the disease. The more severe patients have very high levels of IL-10, IL-6, and TNFα; and fewer CD8+ and CD4+ T cells (9). Previous animal studies have shown that the cytokine storm dampens adaptive immunity against COVID-19 infection (10).

Chen et al. (11) have recently demonstrated immunological differences between moderate vs. severe COVID-19 patients. They demonstrate that CD8+ and CD4+ T cell numbers decrease significantly in patients with severe COVID-19. In patients with moderate COVID-19 the concentrations of IL-10, IL-6, and TNFα are within normal limits, and in the most severe patients they are very high. These cytokines are produced by macrophages and are involved in the cytokine storm (12). The cytokine storm magnifies the danger signal of the virus invasion, but also leads to destructive inflammation and host cell damage (13). In turn, the components released from damaged cells, particularly from stressed mitochondria, including mitochondrial DNA, cardiolipin, cytochrome C and also segments of nuclear DNA are recognized as damage associated molecular patterns by intra and intercellular immune molecules including toll-like receptors 4,7, and 9. Cyclic GMP-AMP synthase triggers a further large-scale proinflammatory cytokine release known as the “secondary cytokine storm”. If this vicious cycle is not interrupted, it results in widespread apoptosis, pyroptosis, and necrosis even of non-infected cells (13).

COVID-19 infection may attack the melatonin synthetic pathway resulting in reduced melatonin levels at a time when melatonin is most needed (14). The uncontrolled innate immune response promotes a massive inflammatory reaction and causes irreversible tissue damage and mortality. Melatonin is a potent antioxidant and immune regulator that not only suppresses oxidative stress but also controls the innate immune response and promotes the adaptive immune response (15, 16). The pineal gland produces and maintains the concentration of melatonin in the blood. The melatonin synthesized in the pineal gland is <5% of the total melatonin produced. The melatonin produced in the mitochondria is not discharged into the circulation, but is used by the cells that produce it (15). If patients do not generate sufficient amounts of melatonin their health status is likely compromised (16).

Autophagy plays an important role both in the antiviral defense responses and in the promotion of the different stages of the viral life cycle. The fact that melatonin is a regulator of autophagy due to its properties as a potent antioxidant and suppressor of endoplasmic reticulum stress suggests a potential beneficial role for this molecule in the management of some viral infections (17). Viruses, including Ebola, dengue, encephalomyocarditis, Venezuelan equine encephalitis, rabbit hemorrhagic disease, human papilloma, and inter alia, have demonstrated the success of melatonin in protecting against viral infections. There is no evidence that melatonin is viricidal but rather it reduces the severity of these infections (18–21). Melatonin's beneficial effects derive from its anti-inflammatory properties, free radical scavenging activity, and immunomodulatory functions.

## Use of Melatonin for Treatment of COVID-19 in the Population

Pharmaceutical laboratories are competing to identify vaccines for COVID-19. According to Benjami Neuman, a virologist, “it is difficult to immunize against the coronavirus, since there has never been a successful human vaccine against any member of the coronavirus family” (22).

The cytokine storm leads to acute cardiac injury, acute respiratory distress syndrome, and infection, leading to generalized sepsis and multisystem failure, which may lead to death (11, 12). Thus, preventing the cytokine storm may be key for the treatment of COVID-19 infected patients. Since there is a lack of effective therapies and immunological treatments may be insufficient, melatonin, owing to its multiple actions as summarized by Zhang et al. (8), may have beneficial effects in preventing or attenuating the cytokine storm and reducing morbidity and mortality from this disease.

### Melatonin, the Elderly & COVID-19

A relationship between melatonin and aging has been suggested, due to a decrease in the concentration of nocturnal melatonin levels in the elderly (23). It has been hypothesized that melatonin can prolong life (24). The relationship of melatonin with aging involves three potential mechanisms: first, melatonin is a key molecule in regular circadian rhythms (25); second, melatonin prevents cardiolipin peroxidation and regulates the synthesis of mitochondrial proteins (26); finally, melatonin secreted by leukocytes exerts a powerful immunomodulatory function (24).

Wu and colleagues have recently shown that advanced age is a poor prognostic factor in patients with COVID-19. This is due to the fact that in the elderly their immune response and physiological functions are decreased as a result of age; therefore, they are more likely to develop severe pneumonia due to COVID-19 (25). Recent studies have shown that high levels of melatonin in the blood play a positive role in health and aging (27). These findings support a rationale for melatonin use in elderly suffering with COVID-19.

### Melatonin, Medical Comorbidities & COVID-19

Aging is a biological process that contributes to an increase in cardiovascular morbidity and mortality. In the HEIJO-KYO cohort (cohort of elderly Japanese patients), urinary excretion of melatonin was associated with reduced nocturnal systolic blood pressure, independent of other cardiovascular risk factors. More precisely, an increase in urinary melatonin excretion from 4.2 to 10.5 μg caused a 2 mmHg decrease in nocturnal systolic blood pressure. Patients who took melatonin at a dose of 2–5 mg/day for 7–90 days uniformly showed a reduction in night-time blood pressure (28, 29).

Obesity is a risk factor for cardiovascular disease. In different studies, melatonin has been shown to have anti-obesity effects (30, 31). Taking melatonin reduces intra-abdominal visceral fat deposition and body weight. Its antiobesogenic effects are believed to be due to two processes: regulation of energy reserves and a relationship with the physiological processes of wakefulness/sleep rhythm (32).

Diabetes is a risk factor for the development of cardiovascular diseases. Several studies have shown a functional interaction between insulin and melatonin, showing that diabetic subjects have a lower concentration of melatonin (33). Furthermore, decreased blood melatonin levels have been documented in patients with insulin resistance or glucose intolerance (34). The results of several studies suggest that low melatonin production is associated with an increased risk of cardiovascular disease (35–37).

Several studies have shown that 75% of COVID-19 patients have 1 or 2 medical comorbidities (38, 39). Other authors have reported that patients with hypertension, obesity, and diabetes are more likely to develop more severe COVID-19 infection, including death (40). The occurrence of heart failure and myocardial infarction is plausible in these patients. The immune system of these patients is altered, with a reduced immune response (40). Furthermore, obesity contributes to various chronic diseases; decreased immunity and subsequently an increased risk of infection (41). Therefore, medical comorbidities are a risk factor for a poor prognosis for patients with COVID-19. Published reports routinely show that melatonin reduces the consequences of the comorbidities in patients with COVID-19.

### Melatonin in COVID-19 Outbreak: Prevention in the Population (Elderly & Medical Comorbidities)

While physiological melatonin concentrations in biological fluids oscillate between 10^−10^ and 10^−11^ M range, a concentration of 10^−5^ M is required to elicit significant pharmacological effects (42). Melatonin protects against cellular damage induced by reactive oxidative species, thus justifying the need of a more generous supplementation of exogenous melatonin in life-threatening pathologies. Oral melatonin use by humans is generally considered safe, with minor side effects including headache, drowsiness, etc. (43). To date, the best dose of melatonin in older adults has not been determined, as its endogenous levels are subject to altered pharmacokinetics. This causes intra-individual variability (44). In a meta-analysis of 50 studies, some of which were not blinded, the efficacy of oral melatonin administration (1–20 mg) was evaluated caused only a few minor adverse side effects, commonly fatigue, and drowsiness (45).

**Figure 1:**
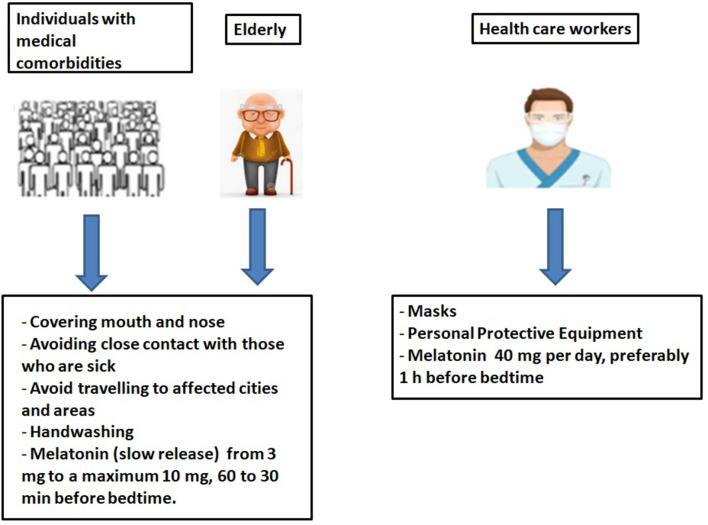
Prevention of COVID-19 infections in the elderly, in individuals with comorbidities and in health care workers.

In elderly patients with medical comorbidities, treatment with melatonin is beneficial, as it strengthens the immune response. We suggest a daily dose of ~3 mg to a maximum of 10 mg, 30–60 min before bedtime to better simulate the normal physiological circadian rhythm of melatonin (Figure 1). Furthermore, it may be beneficial in people who are at high risk of contracting COVID-19 infection, local health workers, where preventive treatment with melatonin would favor maximizing the immune response, along with anti-inflammatory and antioxidant effects. A daily dose of roughly 40 mg or higher would not seem an inappropriate amount (Figure 1).

### Melatonin in COVID-19 Outbreak: Treatment in the Hospital

The world is now facing a pandemic of COVID-19, for which no proven specific therapies are available, other than supportive care. In China, France, Spain, and Italy, a large number of patients have received compassionate use therapies. These therapies have been mostly given without controls, except for a few randomized trials initiated in China, and more recently in the US (46). In the 2014 Ebola outbreak, a randomized clinical trial was implemented and successfully launched during the outbreak; however, it was too late for the trials to be completed in time to be helpful to the currently or soon-to-be infective population (47). In our view, this tragedy cannot be repeated. The COVID-19 pandemic is catastrophic, even though different countries have implemented strict control measures.

Good medical practice requires the physician to use legally available medications according to knowledge-based evidence. If physicians use a product for an indication that is not currently approved, they must base its use on sound scientific reasons and sound medical evidence. Melatonin should be considered a treatment option for this deadly disease.

Melatonin has been shown to be clinically useful in sepsis (43), where the clinical features parallel those of COVID-19 viral infection; moreover, melatonin has been demonstrated to relieve many of the symptoms of other viral infections (17–21, 48). Given the current worldwide situation and in consideration of evidence-based medicine, the efficacy of melatonin and its high pharmacological safety profile supports its use in the treatment of infectious diseases, such as COVID-19. Melatonin can also be useful as a supplement with other treatment (hydroxychloroquine/chloroquine, lopinavir, remidisvir, etc).

Our research group has extensive experience in the use of melatonin in the context of cardiovascular physiology. Melatonin can be administered at a total dose of at least 120 to 1,000 μg/kg/subject weight and intravenously with a high safety profile (36, 37). An aggressive approach is required to prevent coronavirus disease progression and mechanical ventilation. Nordlund and Lerner (49) published a report years ago in which he gave humans one gram of melatonin daily for a month with no untoward effect. Melatonin has a large safety margin without serious adverse effects.

**Figure 2:**
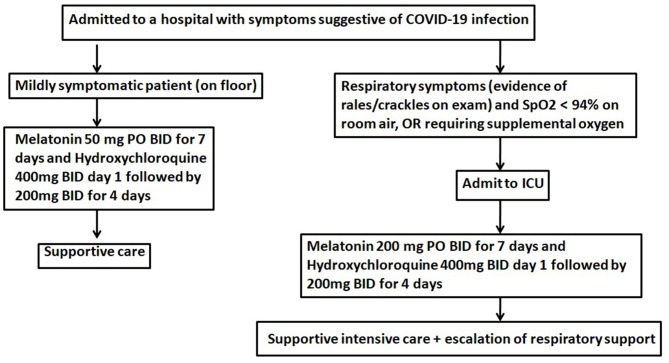
Therapeutic algorithm for use of melatonin in patients with COVID-19. Melatonin will likely reduce the toxicity of chloroquine and increase its efficacy. BID, twice daily; PO, per oral.

Our doses are based in an article recently published by Ramos et al. (50). The authors demonstrated that when we extrapolated effective animal doses to human for a 70 kg adult, the results ranged from 19 to 1,527 mg per day. As there is no time or clinical trials to test the efficacy of melatonin at different concentrations, we suggest the use of melatonin (100 or 400 mg per day) as an adjunct, especially if no efficient direct anti-viral treatment is available (Figure 2).

## Conclusion

The COVID-19 pandemic has infected hundreds of thousands and killed tens of thousands of individuals worldwide. Time is not a luxury that this crisis has. The high mortality is caused by the uncontrolled innate immune response and destructive inflammation. Melatonin is a molecule that negatively regulates the overreaction of the innate immune response and excess inflammation, promoting adaptive immune activity. Moreover, the indole is an endogenous molecule, produced in small amounts, whose synthesis diminishes with increased age. These finding, together with those recently summarized by Anderson and Reiter (51) and Zhang et al. (8), support the use of melatonin in patients with COVID-19. We agree with the suggestion by those authors that melatonin should be given consideration for prophylactic use or treatment alone or in combination with other drugs, and propose a therapeutic algorithm for use in patients. Melatonin is readily available, can be easily synthesized in large quantities, is inexpensive, has a very high safety profile and can be easily self-administered.

## Author Contributions

RR: conceptualization, writing-review, and editing. PA-G and PM: writing-review and editing. AD-R: resources, writing original draft, review, and editing. All authors listed have made a substantial, direct and intellectual contribution to the work, and approved it for publication.

### Conflict of Interest

The authors declare that the research was conducted in the absence of any commercial or financial relationships that could be construed as a potential conflict of interest.
